# KMT5B is required for early motor development

**DOI:** 10.3389/fgene.2022.901228

**Published:** 2022-08-12

**Authors:** Jason Hulen, Dorothy Kenny, Rebecca Black, Jodi Hallgren, Kelley G. Hammond, Eric C. Bredahl, Rochelle N. Wickramasekara, Peter W. Abel, Holly A. F. Stessman

**Affiliations:** ^1^ Department of Pharmacology and Neuroscience, School of Medicine, Creighton University, Omaha, NE, United States; ^2^ Department of Exercise Science, College of Arts and Sciences, Creighton University, Omaha, NE, United States; ^3^ Molecular Diagnostic Laboratory, Boys Town National Research Hospital, Omaha, NE, United States

**Keywords:** H4K20, KMT5B, SUV420H, neuromuscular junction, neuromuscular development, histone methylation, hypotonia, hypertrophy

## Abstract

Disruptive variants in lysine methyl transferase 5B (KMT5B/SUV4-20H1) have been identified as likely-pathogenic among humans with neurodevelopmental phenotypes including motor deficits (i.e., hypotonia and motor delay). However, the role that this enzyme plays in early motor development is largely unknown. Using a *Kmt5b* gene trap mouse model, we assessed neuromuscular strength, skeletal muscle weight (i.e., muscle mass), neuromuscular junction (NMJ) structure, and myofiber type, size, and distribution. Tests were performed over developmental time (postnatal days 17 and 44) to represent postnatal versus adult structures in slow- and fast-twitch muscle types. Prior to the onset of puberty, slow-twitch muscle weight was significantly reduced in heterozygous compared to wild-type males but not females. At the young adult stage, we identified decreased neuromuscular strength, decreased skeletal muscle weights (both slow- and fast-twitch), increased NMJ fragmentation (in slow-twitch muscle), and smaller myofibers in both sexes. We conclude that *Kmt5b* haploinsufficiency results in a skeletal muscle developmental deficit causing reduced muscle mass and body weight.

## Introduction

High-throughput sequencing has identified lysine methyl transferase 5B, *KMT5B* (also known as *SUV4-20H1*), as a high impact neurodevelopmental disorder risk gene in humans (Stessman et al., 2017; Trinh et al., 2019). Primary *KMT5B* patient phenotypes include language delay, intellectual disability, autism spectrum disorder, and motor disorders (i.e., hypotonia and motor delay) (Faundes et al., 2017; Stessman et al., 2017; Trinh et al., 2019). The KMT5B protein catalyzes the dimethylation (me2) of lysine 20 on histone 4 tails (H4K20), which is thought to be the primary substrate for H4K20 trimethylation (me3) by KMT5C (Schotta et al., 2004; Wu et al., 2013). H4K20 methylation affects chromatin conformation, altering chromatin accessibility (Jenuwein and Allis, 2001; Shoaib et al., 2021). H4K20me3 marks are co-localized to pericentric heterochromatin in the genome and are associated with chromatin compaction and gene silencing. Pericentric chromatin aggregation has been positively correlated with myogenic differentiation (Biron et al., 2004; Terranova et al., 2005; Tsang et al., 2010), and previous research has identified a role for KMT5B in the maintenance of the adult muscle satellite cell pool (Boonsanay et al., 2016). These studies strongly support the hypothesis that KMT5B activity contributes to muscle formation and health (Neguembor et al., 2013; Boonsanay et al., 2016). Early skeletal muscle development has not been previously explored in the context of KMT5B deficiencies and was, therefore, the focus of this study. We show that constitutive Kmt*5b* haploinsufficiency in a mouse model results in decreased neuromuscular strength, reduced muscle weight (i.e., muscle mass), and changes in myofiber pathology at both young adolescent and young adult stages of development resembling the motor symptoms noted among the *KMT5B* patient population. These deficits are in addition to the motor reflex and body weight deficits we have previously reported in this model (Wickramasekara et al., 2021). Our results represent data collected from two distinct muscles, the soleus (SOL) and extensor digitorum longus (EDL). Both muscles are located on the lower hind limb but are characterized as slow-twitch vs. fast-twitch, respectively, due to differences in myofiber type composition between the muscles. Given sex differences noted previously (Wickramasekara et al., 2021), we compared our results between both sexes and genotypes. We found that *Kmt5b* heterozygous males were affected earlier and more significantly than females. We propose a model in which motor delays present in *KMT5B* patients are due to changes in skeletal muscle maturation.

## Materials and methods

### Ethical statement

All mouse work was approved and monitored by the Creighton University Institutional Animal Care and Use Committee (IACUC) under protocol numbers 1039 and 1040.

### Mouse model

*Kmt5b* gene-trap mice (Kmt5b^tm1a(KOMP)Wtsi flox−/+^), referred to here as “*Kmt5b* ^*+/GT*^,” are commercially available (KOMP, University of California, Davis, CA, United States). This mouse model contains a LacZ gene trap inserted between exon 4 and exon 5. Mice carrying one copy of the gene trap are considered germline haploinsufficient (Wickramasekara et al., 2021). All experiments were performed blind to mouse genotype.

### RT-qPCR

SOL and EDL skeletal muscles were dissected from mice at 17 days old (P17) and stored in RNA*later* (ThermoFisher Scientific, Waltham, MA, United States). RNA extraction was performed using the RNeasy Fibrous Tissue Mini Kit (Qiagen, Hilden, Germany). Synthesis of cDNA was performed with the iScript cDNA Synthesis Kit (BioRad, Hercules, CA, United States) on a BioRad C1000 Touch Thermocycler followed by quantitative PCR using SsoAdvanced Universal SYBR Green Supermix (BioRad) and a BioRad CFX Connect Real Time System. PrimePCR SYBR Green Assay primers specific for β-Actin (*Actb*; qMmuCED0027505), *Kmt5b* (*Suv420h1*; qMmuCID0014487), and *Kmt5c* (*Suv420h2*; qMmuCID0020579) were sourced from BioRad. Primers for mouse *Gm16066* were designed by and purchased from IDT (Coralville, IA, United States); F-5′-GGCAATACCAGAGGAGAAAGAC-3′and R-5′-CACAGAGAACCCTTGTCTCAAA-3’. The *Gm16066* synthetic control sequence was also purchased from IDT; 5′-TGT​AGG​CAA​TAC​CAG​AGG​AGA​AAG​ACA​GCA​TCG​TTG​TCT​TGG​TTT​GTT​TGA​TTT​TAA​TTA​ATT​AAT​TAA​TTA​ATT​AAT​TAT​TTT​GAG​ACA​AGG​GTT​CTC​TGT​GCC​CT-3′.

### Neuromuscular strength

Mice were held by the tail and allowed to grasp a triangular bar attached to a grip strength meter (Columbus Instruments, Columbus, OH, United States) with their forearm paws. The mouse was pulled *via* the tail in a parallel, opposite direction away from the force meter until the bar was released, and the peak force generated was recorded. Data presented represent an average of three trials per mouse with at least 5 min of rest between trials.

### Neuromuscular junction immunofluorescence

Bilateral SOL and EDL muscles were dissected following perfusion with 4% paraformaldehyde. Muscles were stored in 4% paraformaldehyde at 4°C until further use. Muscles were rinsed in wash solution (2% Triton X-100 in phosphate buffered saline) for 30 min followed by incubation in a blocking solution consisting of 4% bovine serum albumin and 1% Triton X-100 in phosphate buffered saline. Primary incubation was performed overnight in blocking solution at 4°C using mouse IgG antibodies specific for synaptic vesicle glycoprotein 2A (SV2) and neurofilament medium (2H3; Developmental Studies Hybridoma Bank, Iowa City, IA, United States) at concentrations of 0.29 μg/ml and 0.42 μg/ml, respectively. Following primary incubation, muscles were washed three times for 15 min each in wash solution. Secondary incubation was performed in blocking solution for 2 h at room temperature using a goat anti-mouse IgG Alexa Fluor 488 antibody (ThermoFisher Scientific) and tetramethylrhodamine α-bungarotoxin at concentrations of 8.0 μg/ml and 1.5 μg/ml, respectively. Following three final washes for 15 min each, the muscles were placed on slides and cover slipped after adding Vectashield mounting medium (Vector Laboratories, Burlingame, CA, United States). Maximum intensity projection of a Z-stacked image was achieved using a Nikon Ti-E inverted microscope with a Yokagawa Spinning Disc at ×40 magnification. Images were processed using ImageJ 1.52a software (Schneider et al., 2012) with the binaryconnectivity. class plugin (Landini, 2020). Neuromuscular junction images were processed according to previously published methods to obtain total area, discontinuity (1 adjacent pixel), and branch points (3 + adjacent pixels) for both muscle and nerve/synaptic vesicle areas (Pratt et al., 2018).

### Myosin heavy chain immunofluorescence

Mice were sacrificed using CO_2_ asphyxiation and the left SOL and EDL were dissected, frozen in liquid nitrogen, and stored at −80°C. Frozen muscles were embedded in OCT compound (ThermoFisher Scientific), cryosectioned near the mid-belly at 5 μm thickness, and collected onto slides. Frozen sections were thawed to room temperature for 15 min immediately prior to the immunofluorescence procedure. Sections were washed in 0.5% Triton X-100 in phosphate buffered saline for 30 min. Slides were then incubated in a humidified chamber at room temperature with a blocking solution consisting of 20% normal goat serum and 0.5% Triton X-100 in phosphate buffered saline for 1 h. Primary antibodies specific for myosin heavy chain type I (mouse IgG2b), myosin heavy chain IIa (mouse IgG1), myosin heavy chain IIb (mouse IgM) (BA-D5, SC-71, and BF-F3, respectively; Developmental Studies Hybridoma Bank), and laminin (rabbit IgG; Sigma-Aldrich, St. Louis, MO, United States) were prepared in blocking solution at concentrations of 7.625 μg/ml BA-D5, 1.935 μg/ml SC-71, 2.015 μg/ml BF-F3, and 1 μg/ml laminin, and incubated for 1 h at room temperature in a humidified chamber. After a 30-min wash, secondary antibody incubation was performed with 20 μg/ml goat anti-mouse IgG2b Alexa Fluor 647, 2.6 μg/ml goat anti-mouse IgG1 Alexa Fluor 546, 5.7 μg/ml goat anti-mouse IgM Alexa Fluor 488, and 4.0 μg/ml goat anti-rabbit IgG Alexa Fluor 405 (ThermoFisher Scientific) in blocking solution for 2 hours at room temperature in a humidified chamber. Following a final 30-min wash, sections had Vectashield medium added and were cover slipped. Imaging was achieved using a Nikon Ti-E inverted microscope with a Yokagawa Spinning Disc at ×10 magnification. Images were processed and analyzed using ImageJ 1.52a software (Schneider et al., 2012) for total area and immunofluorescence area. The number of myofiber cells for each antibody was counted manually and blinded.

### Data analyses

All statistical calculations were performed using GraphPad Prism version 8.3.0 for Windows (GraphPad Software, San Diego, CA, United States). All primary data were analyzed using two-way ANOVAs with independent variables of genotype and sex. Mouse body weight was analyzed using a two-way (genotype x age) ANOVA with repeated measures. Where indicated, a post hoc test, Šídák’s test, Tukey’s multiple comparisons test, or Fisher’s LSD, was used to further identify specific relationships. Pearson’s correlation tests were used to test specific pairs of continuous variables where noted. Genotype effects of the mother (i.e., dam) were analyzed using three-way ANOVAs with independent variables of dam genotype, mouse genotype, and sex.

## Results

### Kmt5b HET mice exhibit neuromuscular strength deficits consistent with human hypotonia

**FIGURE 1:**
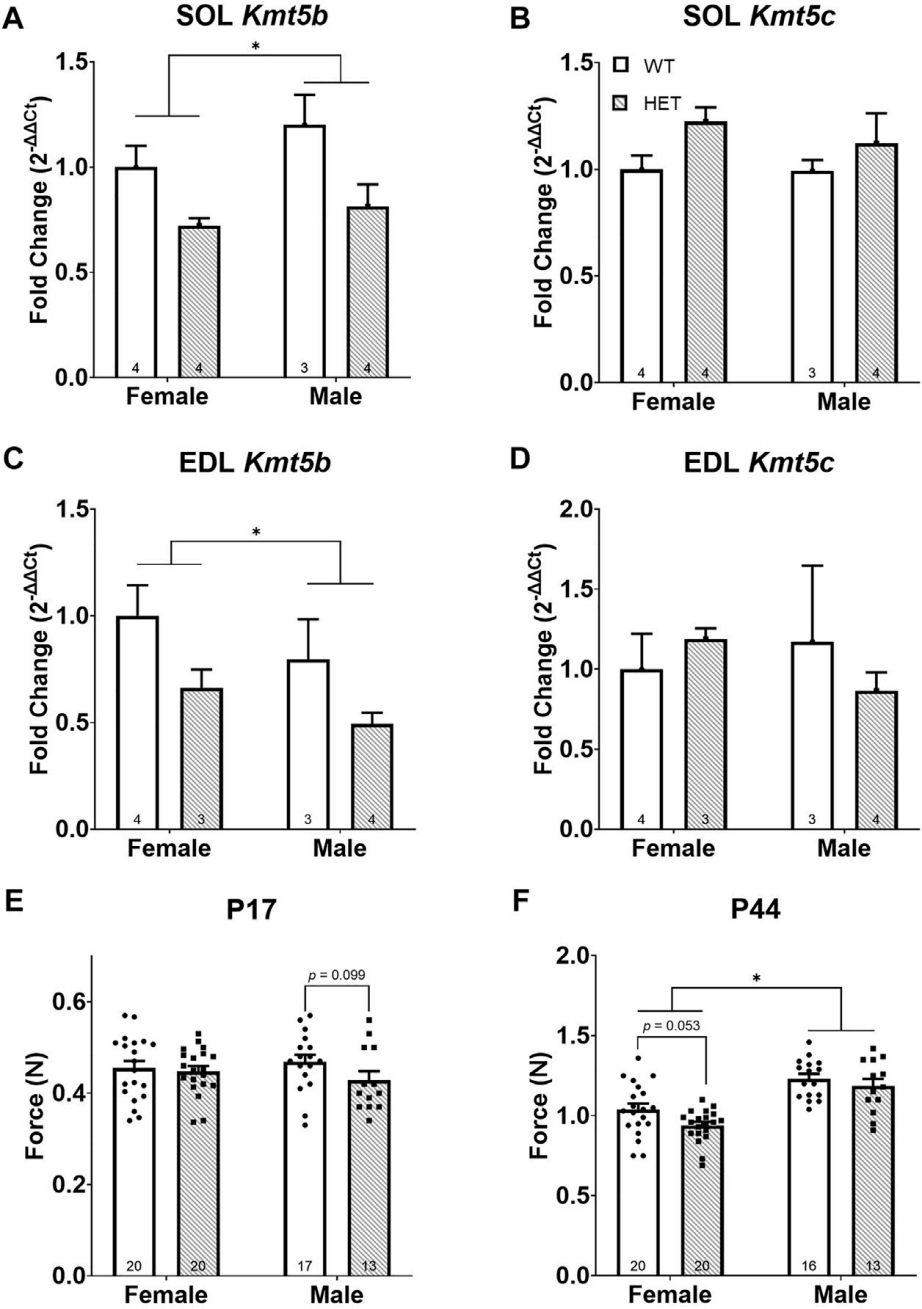
*Kmt5b HET* mice show a progressive strength deficit. Gene expression of *Kmt5b* **(A,C)** and *Kmt5c* **(B,D)** measured by qRT-PCR shown for soleus [SOL; **(A,B)]** and extensor digitorum longus [EDL; **(C,D)]** muscles at postnatal day (P) 17. Results of pull bar strength testing are shown collected at P17 **(E)** and P44 **(F)**. *WT*: dots and open bars; *HET*: squares and grey hatched bars. *p < 0.05; (2-way ANOVA). *p*-values shown are the results of post hoc testing. Error bars show ±SEM; number of biological replicates are shown within each bar.

Motor delays and hypotonia have been frequently reported in humans carrying germline heterozygous disruptive *KMT5B* variation (Trinh et al., 2019; Wickramasekara and Stessman, 2019). Previously, we reported early motor reflex deficits using a LacZ gene trapping allele (Wickramasekara et al., 2021) to mimic germline heterozygous *Kmt5b* loss in mice (*Kmt5b* ^*+/GT*^; referred to as “*HET*”). To better understand the contribution of KMT5B to early muscle development and function, we have further utilized this mouse model to specifically study the effects of *Kmt5b* loss on hindlimb skeletal muscle development. Homozygous *Kmt5b* knockout is perinatally lethal (Schotta et al., 2008; Wickramasekara et al., 2021); therefore, only wild-type (*WT*) and heterozygous (*HET*) genotypes were studied. Quantitative RT-PCR on mRNA extracted from P17 soleus (SOL) and extensor digitorum longus (EDL) hindlimb muscles confirmed that *Kmt5b* is expressed in skeletal muscle during development and that gene trapping on one allele (i.e., *HET*) reduced this expression by approximately half in both sexes, as expected (Figures 1A,C). *Kmt5c* expression showed no changes in expression by genotype or sex (Figures 1B,D).

To compare strength between *WT* and *HET* animals, we performed a neuromuscular “pull bar” strength test (Smith et al., 1995) on postnatal (P) days 17 and P44. These time points were chosen to most closely mimic the neonatal human infant state (Draeger et al., 1987; Butler-Browne et al., 1990) and early sexual maturity, respectively. We observed no significant differences in neuromuscular strength in *HET* mice at age P17 (Figure 1E). At P44, as expected, sex had a significant effect on neuromuscular strength [*F* (1, 65) = 41.14; *p* < 0.0001; Figure 1F] where it was increased in males compared to females. Genotype significantly impacted strength where *HET* mice showed less neuromuscular strength than *WT* mice [*F* (1, 65) = 4.550; *p* = 0.0367]; however, neither sex survived post hoc testing (Figure 1F). Body weight was significantly decreased in both *HET* males [*F* (1, 27) = 36.58; *p* < 0.0001] and females [*F* (1, 38) = 10.80; *p* = 0.0022] compared to their *WT* counterparts (data not shown), consistent with our previous work (Wickramasekara et al., 2021). Neuromuscular strength and body weight were only correlated consistently among *WT* females (at both P17 and P44) but not among males (Supplementary Figure S1). These results supported the hypothesis that *Kmt5b* loss is directly associated with hypotonia (i.e., reduced muscle tone).

### Progressive muscle weakness is accompanied by decreasing muscle weights

Given the known role of KMT5B in adult muscle regeneration (Boonsanay et al., 2016), we hypothesized that the neuromuscular strength deficits observed were due to a muscle-specific requirement for KMT5B in early development. The SOL and EDL muscles were chosen for further study as they are both located on the lower hind limb but represent two distinct skeletal muscle populations; the SOL represents a slow-twitch (type I) muscle and the EDL represents a fast-twitch (type II) muscle.

**FIGURE 2:**
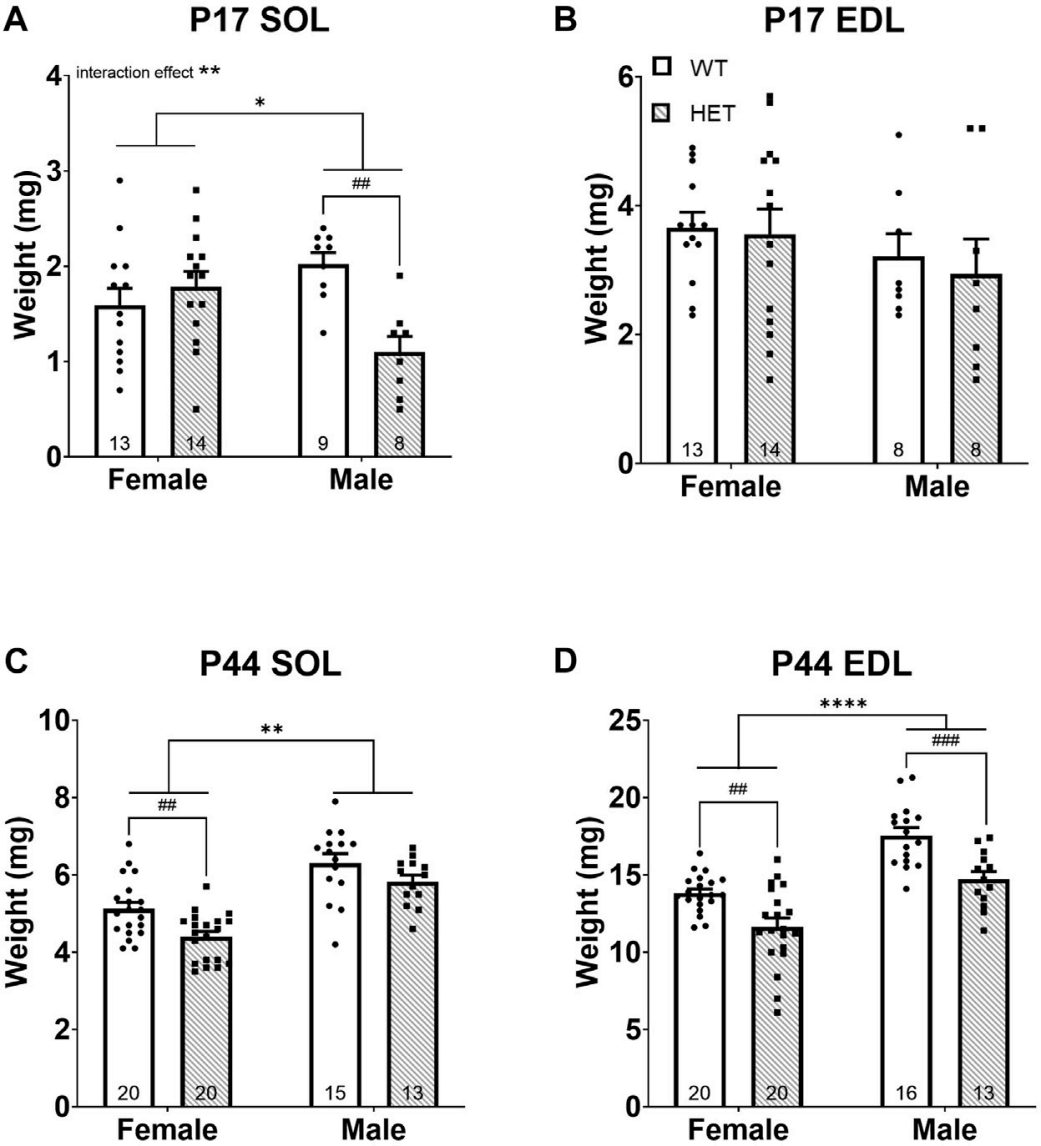
*HET* mice have decreased skeletal muscle weights (i.e., muscle mass). Skeletal muscles weight at ages P17 and P44 for SOL **(A,C)** and EDL **(B,D)** are shown. *p < 0.05; **p < 0.01; ****p < 0.0001 (2-way ANOVA). ##*p* < 0.01; ###*p* < 0.001 (post hoc test; effect of genotype only). Error bars show ±SEM; number of biological replicates are shown within each bar.

At P17, SOL weights were significantly impacted by both genotype [*F* (1, 40) = 4.544; *p* = 0.0392] and an interaction between sex and genotype [F (1, 40) = 10.65; *p* = 0.0023]. Post hoc testing showed that this effect was driven by a significant decrease in SOL weight in male *HET* compared to *WT* animals (*p* = 0.0072; Tukey’s multiple comparisons test; Figure 2A). There were no differences in EDL weight at P17 (Figure 2B). At P44, both SOL [*F* (1, 64) = 10.99; *p* = 0.0015; Figure 2C] and EDL [*F* (1, 65) = 26.65; *p* < 0.0001; Figure 2D] weights were significantly impacted by genotype. In both muscle types, *HET* muscles weighed less than in *WT* animals. This effect survived post hoc testing in females for both the SOL (*p* = 0.0057; Šídák’s test) and EDL (*p* = 0.0018; Šídák’s test; Figures 2C,D) and was even more significant in males for the EDL (*p* = 0.0006; Šídák’s test; Figure 2D). As expected, there was also a significant difference between sexes after puberty (i.e., P44); males had increased muscle weights overall [SOL: *F* (1, 64) = 51.06; *p* < 0.0001; EDL: *F* (1, 65) = 48.89; *p* < 0.0001; Figures 2C,D].

Muscle weight (i.e., lean muscle mass) is a known significant contributor to body weight and was also significantly correlated with body weight in this study (Supplementary Figure S2). Given the significantly reduced body weights in *HET* compared to *WT* mice, we compared muscle weights after normalization to body weight at the time of collection. Normalized P17 SOL muscle weights showed a significant interaction between sex and genotype [*F* (1, 40) = 12.55; *p* = 0.0010; Supplementary Figure S3A driven by strikingly lower muscle weight in *HET* males compared to *WT* males (*p* = 0.0037; Šídák’s test) but no differences in normalized P17 EDL (Supplementary Figure S3B). Therefore, male *HET* SOL muscles were even smaller than would be predicted for their body weight. To determine whether this effect was SOL specific, additional organs (brain, heart, kidney, and liver) were dissected and weighed at P17 (Supplementary Figure S4). While liver (Supplementary Figure S4A), heart (Supplementary Figure S4C), and kidney (Supplementary Figure S4E) weights trended smaller in *HET* males at P17, the liver was most significantly impacted (*p* = 0.0493; Šídák’s test). However, none of these other organs were significantly smaller in *HET* animals after normalization to body weight (Supplementary Figures S4B,D,F,H) suggesting a specific effect on SOL muscles at this age. Interestingly, male *HET* brains were significantly heavier than *WT* animals at P17 after normalization to body weight (Supplementary Figure S4H). There were no significant differences in normalized P44 SOL weights (Supplementary Figure S3C). A significant effect of genotype was noted among normalized P44 EDL muscle weights [*F* (1, 65) = 5.747; *p* = 0.0194; Supplementary Figure S3D]. Female *HET* normalized EDL weights remained modestly lower after post hoc testing (*p* = 0.0445; Šídák’s test). Taken together, these data highlighted decreased neuromuscular strength and muscle mass among *HET* animals affecting both sexes that worsened with age. Loss of slow-twitch (SOL) muscle mass was only apparent in *HET* males at P17 but in all *HET* animals in both muscle types at P44. Fast-twitch (EDL) muscle was more significantly affected as time progressed; this effect was more pronounced among *HET* females.

### KMT5B haploinsufficiency increases neuromuscular junction fragmentation by young adulthood

**FIGURE 3:**
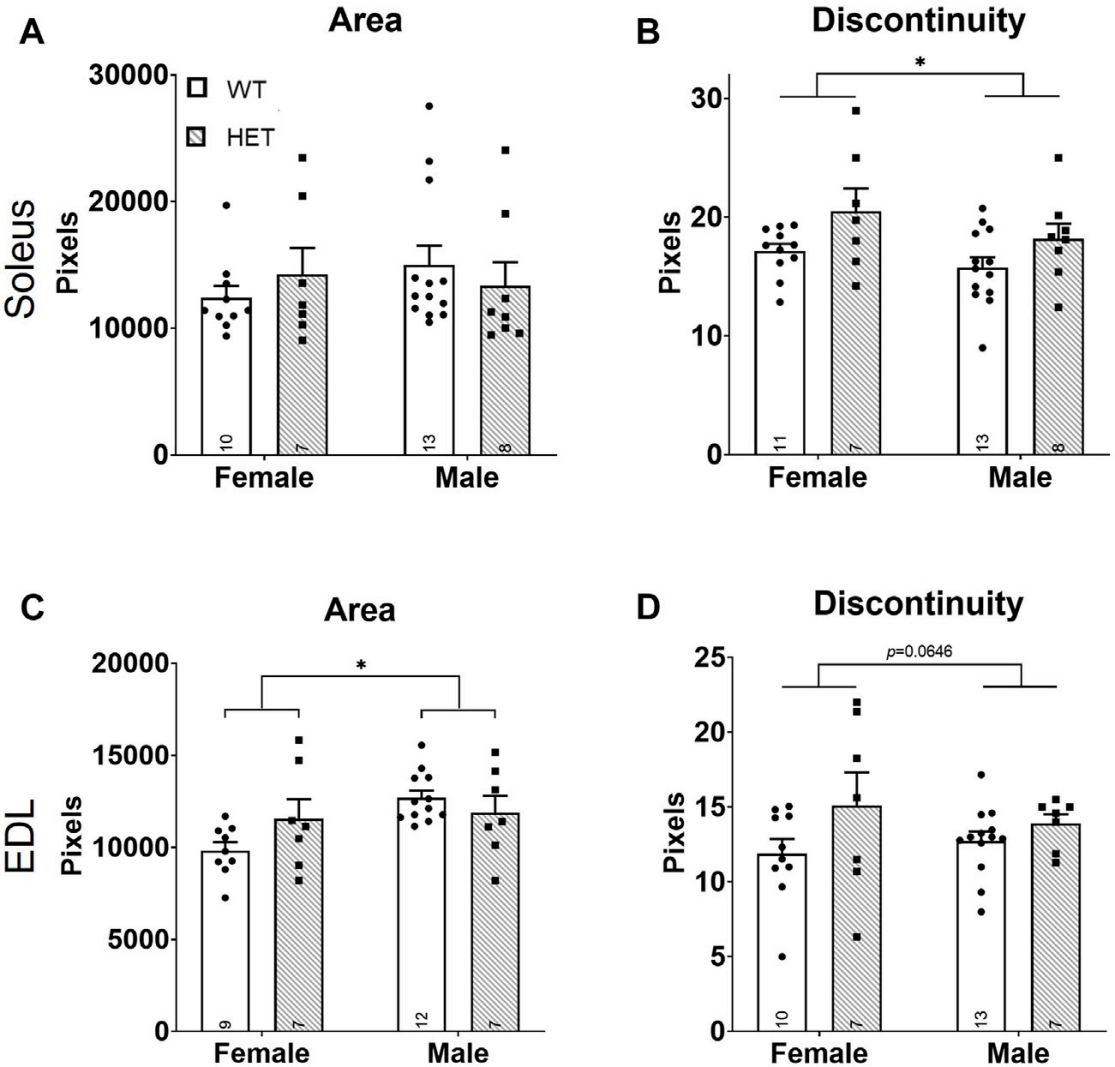
Increased NMJ fragmentation in *HET* skeletal muscles. Whole mount immunofluorescence staining for both nerve/vesicle and skeletal muscle portions of NMJs was performed at P44. Only data for skeletal muscle NMJ portions are shown. Graphs show total area **(A,C)** and calculated NMJ discontinuity **(B,D)** for SOL **(A,B)** and EDL **(C,D)** skeletal muscles. Y-axes of graphs are numbers of pixels. *WT*: dots and open bars; *HET*: squares and grey hatched bars. *p < 0.05 (2-way ANOVA). Error bars show ±SEM; number of biological replicates are shown within each bar.

The major functional unit of skeletal muscle activity is the neuromuscular junction (NMJ). NMJ pathology has been shown to underlie other strength deficits (Souayah et al., 2009; Chai et al., 2011; Klooster et al., 2012; Rudolf et al., 2013; Haddix et al., 2018). Given the strength deficit identified at P44 in our model (Figure 1F), we hypothesized that this was caused by NMJ dysfunction. Both NMJ size and continuity were scored for the nerve/vesicular and muscle portions of the NMJ using immunofluorescence on P44 SOL and EDL muscles (Supplementary Figures S5A,B). NMJ occupancy (defined as the ratio of nerve/vesicular area vs. muscular NMJ area) did not significantly differ in the SOL (Supplementary Figure S5C) or the EDL (Supplementary Figure S5F). No significant differences were identified in nerve/vesicular NMJ area or continuity in either muscle (SOL: Supplementary Figures S5D,E; EDL: Supplementary Figures S5G,H). In the muscle portion of the NMJ (Figure 3), SOL NMJ areas did not differ (Figure 3A), but genotype contributed significantly to discontinuity [*F* (1, 35) = 6.447; *p* = 0.0157; Figure 3B] where *HET* NMJs had increased discontinuity compared to *WT* NMJs. Sex, but not genotype, contributed significantly to NMJ areas in the EDL [*F* (1, 31) = 5.684; *p* = 0.0234; Figure 3C]; area was increased in males compared to females. However, neither sex survived post hoc testing. A trend toward increased discontinuity among *HETs* was noted in the EDL (Figure 3D) but was not statistically significant. These data showed that increased NMJ fragmentation accompanies decreased neuromuscular strength and reduced muscle mass at young adult stages in *HET* mice.

### Fast-twitch myofibers are increased in number but decreased in size in Kmt5b HET mice

Proper motor neuron innervation at the NMJ is required for skeletal muscle myofiber maturation (Jin et al., 2008) and maintenance (Rowan et al., 2012). Myofibers are the predominant structural unit comprising skeletal muscle (Schiaffino and Reggiani, 1985). Changes in myofiber composition have also been linked to strength deficits (Pedemonte et al., 1999; Tanner et al., 2002; Celegato et al., 2006; Chai et al., 2011; Nilwik et al., 2013). Given the increased muscular NMJ fragmentation, reduced neuromuscular strength, and decreased muscle mass among *HET* mice, we analyzed myofiber abundance and size. Both slow- and fast-twitch muscles are composed of type I, IIa, IIx, and IIb fibers at differing ratios (Schiaffino and Reggiani, 1985). Myofiber types can be distinguished through their myosin heavy chain expression (Termin et al., 1989).

**FIGURE 4:**
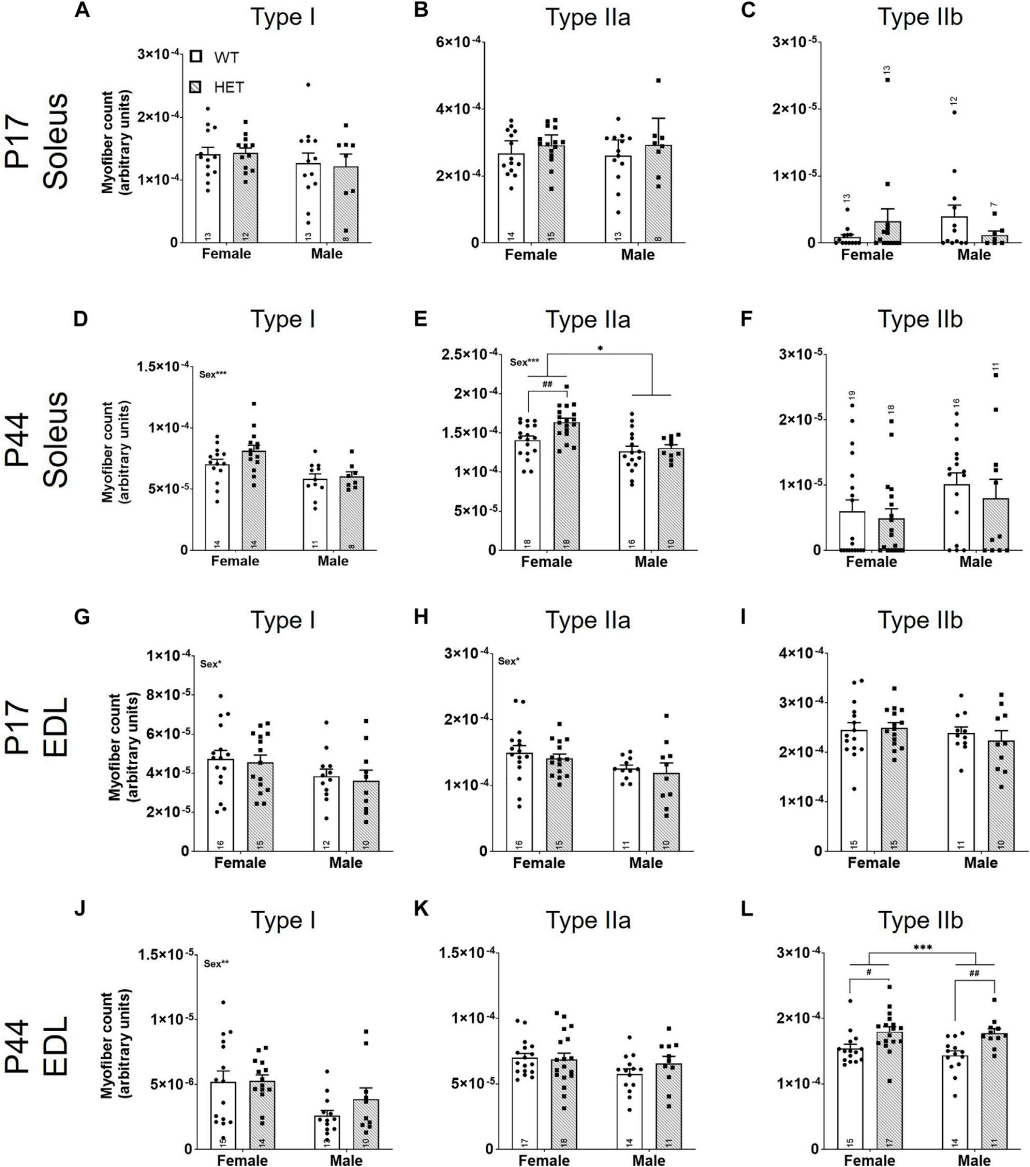
Fast-twitch myofiber counts are increased in *HET* muscles at P44. Myofiber types (I, IIa, and IIb) were scored using immunofluorescence. Individual myofibers per type were counted and normalized to the total area of the section (represented as myofiber count; y-axes). Graphs show each myofiber type in males and females from P17 SOL **(A**–**C)**, P44 SOL **(D**–**F)**, P17 EDL **(G**–**I)**, and P44 EDL **(J**–**L)**. *WT*: dots and open bars; *HET*: squares and grey hatched bars. *p < 0.05; **p < 0.01; ***p < 0.001 (2-way ANOVA). #*p* < 0.05; ##*p* < 0.01 (post hoc test; effect of genotype only). Effects of sex by 2-way ANOVA testing are shown as text in the upper left of the panel. Error bars show ±SEM; number of biological replicates are shown within each bar.

We first quantified the relative myofiber number for myofiber types I, IIa, and IIb in SOL and EDL muscles (Figures 4A–L). For the slow-twitch SOL muscle (Figures 4A–F), there were no differences in myofiber number at P17 (Figures 4A–C). In young adult animals (P44; Figures 4D–F), there was a significant effect of sex on both type I [*F* (1, 43) = 13.85; *p* = 0.0006; Figure 4D] and type IIa [*F* (1, 58) = 17.16; *p* = 0.0001; Figure 4E] myofibers; males had fewer of these myofibers than females. There was also a significant effect of genotype on type IIa myofiber number [*F* (1, 58) = 5.622, *p* = 0.021; Figure 4E]. Post hoc testing showed that this effect was driven by an increase in type IIa fibers in *HET* compared to *WT* females (*p* = 0.0051; Šídák’s test). For the fast-twitch EDL muscle (Figures 4G–L), there was a significant effect of sex on type I [*F* (1, 49) = 4.247; *p* = 0.0447; Figure 4G] and type IIa [*F* (1, 48) = 5.118; *p* = 0.0282; Figure 4H] myofiber numbers at P17 and type I fibers at P44 [*F* (1, 48) = 8.712; *p* = 0.0049; Figure 4J] where, again, male counts were decreased compared to females. Genotype contributed significantly to type IIb myofiber number at P44 [*F* (1, 53) = 17.17, *p* = 0.0001; Figure 4L]. Šídák’s post-hoc analysis identified significantly increased IIb myofiber numbers in both female and male *HET* mice compared to *WT* (*p* = 0.018 and *p* = 0.0054, respectively).

The total fluorescent signal for each myofiber marker was collected and normalized to the total pixel area of the section to identify the percent area for each myofiber type (Supplementary Figures S6A–L). Significant effects of sex were identified for P44 SOL type I [*F* (1, 43) = 4.475; *p* = 0.0402; Supplementary Figure S6D] and IIb [*F* (1, 59) = 9.624; *p* = 0.0029; Supplementary Figure S6F] myofibers and P44 EDL type I [*F* (1, 47) = 11.61; *p* = 0.0014; Supplementary Figure S6J] and IIa [*F* (1, 55) = 5.525; *p* = 0.0224; Supplementary Figure S6K] myofiber areas. A significant interaction between sex and genotype was noted in P17 SOL type IIb myofibers [*F* (1, 42) = 4.107, *p* = 0.0491; Supplementary Figure S6C) that was driven by an increase in type IIb myofibers in female *HET* compared to *WT* (*p* = 0.0439; post hoc uncorrected Fisher’s LSD). Genotype significantly affected the percent area of P44 EDL type IIb [*F* (1, 53) = 6.323; *p* = 0.0150; Supplementary Figure S6L] myofibers; however, this result did not survive post hoc testing. Both cell count and percent area by myofiber type were used to calculate a myofiber relative ratio for each by sex and genotype (Supplementary Figure S7). While myofiber compositions were as expected (e.g., EDL with a majority of fast-twitch and SOL with few fast-twitch myofibers (Agbulut et al., 2003); Supplementary Figures S7B,C), no significant differences among relative proportions [either by count (Supplementary Figure S7B) or area (Supplementary Figure S7C)] were identified between genotype or sex.

**FIGURE 5:**
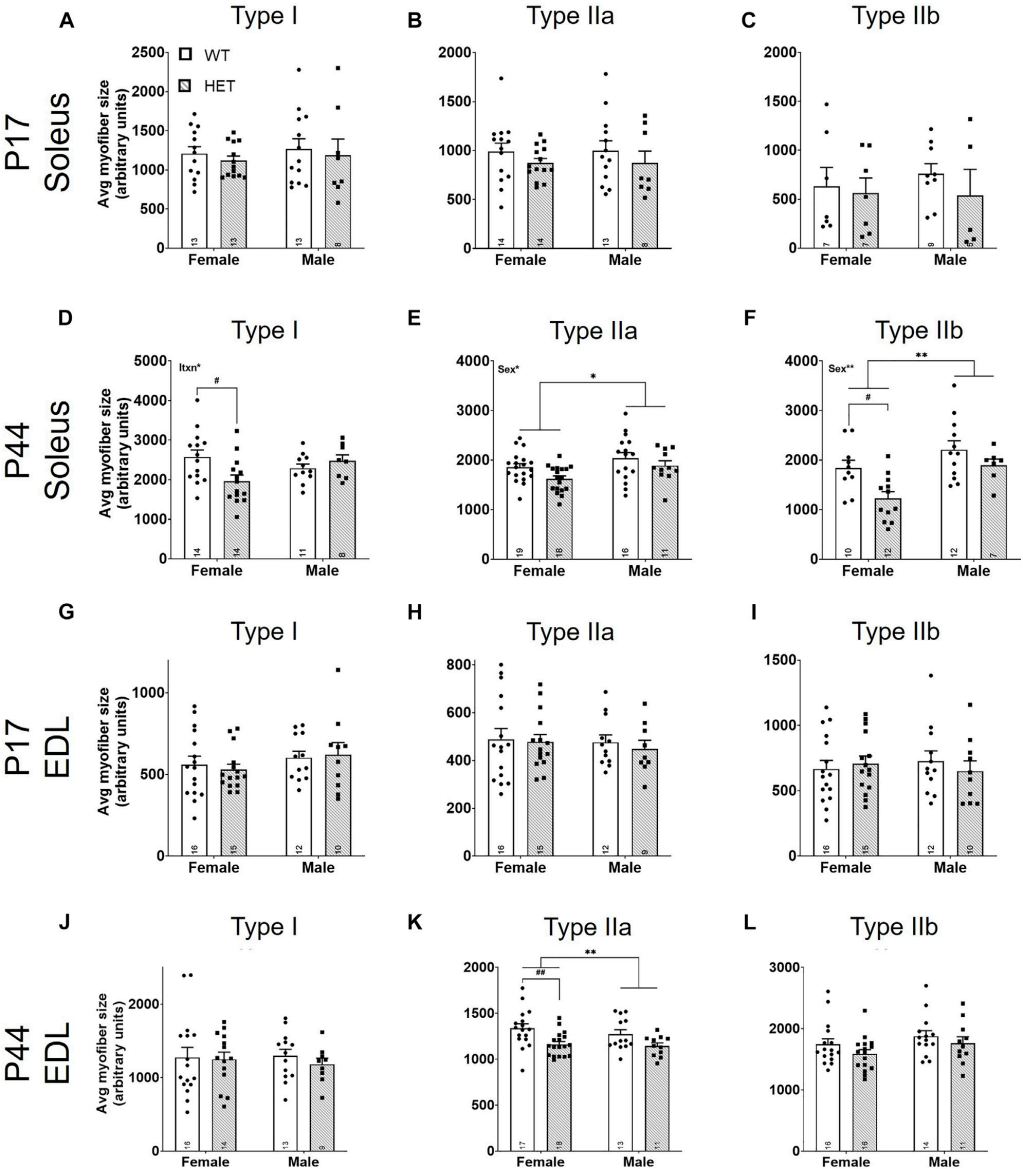
Average fast-twitch myofiber size is decreased at P44. For myofiber types (I, IIa, and IIb), average size was scored using the total myofiber type area by immunofluorescence/myofiber type count (Figure 4; y-axes). Graphs show results of each myofiber type in males and females from P17 SOL **(A**–**C)**, P44 SOL **(D**–**F)**, P17 EDL **(G**–**I)**, and P44 EDL **(J**–**L)**. *WT*: dots and open bars; *HET*: squares and grey hatched bars. *p < 0.05; **p < 0.01 (2-way ANOVA). #*p* < 0.05; ##*p* < 0.01 (post hoc test; effect of genotype only). Effects of sex or an interaction (itxn) between sex and genotype by 2-way ANOVA testing are shown as text in the upper left of the panel. Error bars show ±SEM; number of biological replicates are shown within each bar.

Using the percent area for each myofiber type and the myofiber count for each section, we calculated an average size for each myofiber type (Figures 5A–L). There were no significant differences between myofiber sizes in the SOL (Figures 5A–C) or the EDL (Figures 5G–I) at P17. At P44, there were significant effects of sex on the size of SOL type IIa myofibers [*F* (1, 60) = 6.569; *p* = 0.0129; Figure 5E] and IIb myofibers [*F* (1, 37) = 10.30; *p* = 0.0027; Figure 5F]; male myofibers were larger. SOL type I myofibers had a significant interaction between sex and genotype [*F* (1, 43) = 6.089; *p* = 0.0177; Figure 5D]. By post hoc testing, females had significantly smaller average SOL type I myofiber sizes in *HET* mice compared to *WT* mice (*p* = 0.0266; Šídák’s test). Genotype contributed significantly to average SOL type IIa [*F* (1, 60) = 4.995; *p* = 0.0292; Figure 5E], SOL type IIb [*F* (1, 37) = 8.260; *p* = 0.0067; Figure 5F], and EDL type IIa myofiber sizes [*F* (1, 55) = 12.00, *p* = 0.0010; Figure 5K]. Šídák’s post-hoc analyses identified that female *HETs* had significantly decreased SOL type IIb (*p* = 0.0144) and EDL type IIa (*p* = 0.0057) myofiber sizes compared to *WT* mice.

## Discussion

In this study, we explored the pathological basis for motor deficits that have been identified in human patients carrying heterozygous disruptive *KMT5B* variants (Stessman et al., 2017; Trinh et al., 2019) and in a mouse model of germline *Kmt5b* haploinsufficiency (Wickramasekara et al., 2021). *Kmt5b HET* mice showed a progressive decline in neuromuscular strength from adolescence (P17) to young adulthood (P44). This was underscored by reduced skeletal muscle mass that affected slow-twitch (SOL) at a younger age than fast-twitch (EDL) muscles. This decreased muscle mass likely contributes to the decreased body weights that we have reported previously (Wickramasekara et al., 2021) and also confirmed in this study. Analysis of the NMJ revealed increased fragmentation in *HET* animals, particularly in the slow-twitch SOL. A detailed analysis of skeletal muscle myofiber composition identified increased myofiber counts by young adulthood; however, these myofibers were overall smaller in *HET* compared to *WT* animals. These data highlight an effect of germline *Kmt5b* genotype on the size, structure, and function of skeletal muscle.

Skeletal muscle growth occurs through three primary mechanisms, 1) formation of new cells (myogenesis), 2) merging of myonuclei to growing myofibers (accretion), and 3) increasing the size of cells through increased protein synthesis and decreased degradation (hypertrophy). Myogenesis happens exclusively before birth in mice. Myofiber numbers are considered fixed during postnatal life except in cases of degradation without regeneration (White et al., 2010). The scaffold for adult skeletal muscle is formed during primary myogenesis (embryonic days E10.5–E12.5) from the fusion of Pax3^+^ myocytes expressing exclusively slow-twitch markers. Secondary myogenesis, occurring from E14.5–17.5, is achieved through the fusion of Pax7^+^ progenitor cells to the primary scaffold (Lyons et al., 1990; Horst et al., 2006). This is when fast-twitch markers begin to be expressed, and motor neurons innervate the developing muscles forming NMJs (Slater, 1982); innervation dictates myofiber type moving forward. There is currently no evidence that loss of one germline copy of *Kmt5b* significantly disrupts primary or secondary myogenesis as all muscles analyzed at P17 expressed both the myofiber types and ratios expected. Further, when not corrected for tissue area, myofiber counts were not different between *WT* and *HET* animals at P17 or P44 (not shown) suggesting that the total initial number of myofibers required are present in the *HET* condition.

Accretion occurs postnatally and is often linked with hypertrophy to grow muscle size; all muscle weights in our model were significantly reduced in *HET* animals at P44. Accretion involves the fusion of muscle satellite cells (i.e., myonuclei) with myofibers to support the increased capacity of protein synthesis required for hypertrophy. KMT5B has been previously linked to increased mouse muscle turnover in adulthood due to changes in the satellite cell population (Neguembor et al., 2013; Boonsanay et al., 2016), a specific cell population which we did not directly analyze in this study. In our model, there was no evidence of increased degenerating/regenerating myofibers in *HET* compared to *WT* tibialis anterior muscle sections collected at P44 (not shown). However, newly regenerated myofibers are known to initially express type II (fast-twitch) markers, and specific slow motor neuron input is required to further switch to the type I (slow-twitch) myofiber program (Jerkovic et al., 1997). In other muscle pathologies where myofiber turnover is increased, increased degeneration of myofibers drives fragmentation of the NMJ (Li and Thompson, 2011; Pratt SJ et al., 2015; Haddix et al., 2018). We observe increased yet smaller type II myofibers and increased NMJ fragmentation in SOL muscles of *HET* mice at P44 (young adulthood). While these small type II myofibers could represent newly regenerated myofibers, we believe it more likely that proper developmental accretion/hypertrophy requires KMT5B activity. However, it has been shown that specific *Kmt5b* knockout in Pax7^+^ skeletal muscle progenitor cells produces no obvious growth or clinical abnormalities (Boonsanay et al., 2016) suggesting that KMT5B’s effects on skeletal muscle development could be imparted from outside the skeletal muscles themselves.

Two opposing pathways are known to regulate muscle hypertrophy where growth is stimulated by the insulin-like growth factor 1— phosphoinositide-3-kinase–Akt/protein kinase B—mammalian target of rapamycin (IGF1—PI3K—Akt/PKB—mTOR) pathway and opposed by myostatin (TGFβ superfamily)—Smad3 signaling (Egerman and Glass, 2014). Growth hormone (GH), secreted from the anterior pituitary gland, stimulates the production of IGF1 (Oberbauer, 2013), highlighting a strong force external to the skeletal muscle that is required for growth and maturation. While most IGF1 in the body is expressed from the liver and secreted into circulation (endocrine), target tissues expressing GH receptors including skeletal muscle also express IGF1 locally (autocrine/paracrine) (Wang et al., 2013). Germline knockout of IGF1 or GH receptor in mice results in significant growth deficiencies (Coschigano et al., 2000; Liu et al., 2000). Interestingly, very similar phenotypes to those presented here–reduced muscle and body size and increased yet smaller type II fibers—have been identified in the GH receptor knockout mouse model (Sotiropoulos et al., 2006; Mavalli et al., 2010) supporting our new hypothesis that KMT5B is involved in the anabolic regulation of muscle hypertrophy.

It is interesting that both the sexes and different muscle types appear to respond differently to *Kmt5b* haploinsufficiency. Prior to the onset of puberty (i.e., P17), we noted specific effects on slow-twitch SOL muscles in *HET* males. We have previously shown at this age that *HET* males are also more impacted by body weight and motor reflex changes than *HET* females (Wickramasekara et al., 2021). In this study, the SOL weight deficit was diminished by P44 even though male *HET* body weight remained significantly lower than *WT* males, suggesting a SOL developmental delay in males. Testosterone is a potent anabolic stimulant of muscle hypertrophy, and increases in testosterone are required for sexual maturity. In male mice, testosterone increases in two waves, the first from P25-P30 and the second from P45-P50 (Barkley and Goldman, 1977). Indeed, many motor delays/deficits in human children with neurodevelopmental disorders improve with age (Ming et al., 2007). Whether this is related to the expression of androgens in certain cases or the result of interventional physical and occupational therapies remains unknown (Ming et al., 2007). Adult muscle effects were often more pronounced in females than males in our model. *HET* female mice in this study appeared to develop normally until puberty after which they presented muscle phenotypes (P44) reinforcing that KMT5B is also involved in maintaining adult muscle health, which has also been proposed by others (Neguembor et al., 2013; Boonsanay et al., 2016).

Still other mechanisms may contribute to the phenotypes observed in our model. For example, a recent publication reported decreased body fat in tissue-specific *Kmt5b*/*Kmt5c* double knockout mice (Pedrotti et al., 2019). Decreased intramuscular fat stores in skeletal muscle may contribute to the decreased skeletal muscle weights observed in male *HET* mice. We have previously reported an effect of litter on motor performance in neonatal mice (Wickramasekara et al., 2021). Neonatal nutrition is known to contribute to total body weight and muscle maturation (Glore and Layman, 1983; Miller et al., 1984; Nemeth et al., 1992; O'Connor et al., 2003). Particularly prior to weaning, litter/litter size can contribute to resource acquisition. There were no significant interactions between litter and genotype in this study. However, we did not directly measure food intake at any age in this study. Effects of litter can also be caused by the genotype of the dam (not controlled for in this study). There was no significant effect of dam genotype on neuromuscular strength (Supplementary Figures S8A,B), muscle weight (Supplementary Figures S8C–F), or mouse body weight (Supplementary Figures S8G,H). We conclude that while variability between litters existed in each experiment, the relationship between *Kmt5b* genotype and neuromuscular strength, body weight, and muscle weights remains fixed. Finally, it is possible that the LacZ gene trap affects the expression of neighboring genes compounding this phenotype, such as the predicted lncRNA, *Gm16066*, transcribed from the opposite strand. The expression pattern and function of this lncRNA are largely unknown. We found that *Gm16066* was not expressed in SOL, EDL, or liver (data not shown) by qRT-PCR in these mice; therefore, this is not likely a confounding variable.

Muscle development in humans and mice is similar; however, there are some notable differences. In humans, embryonic muscle development occurs over three myogenesis stages in which primary, secondary, and tertiary myotubes form (Draeger et al., 1987). Around birth, the majority of myofibers in human skeletal muscle are adult myofiber types (Butler-Browne et al., 1990; Schiaffino et al., 2015), as opposed to mice which will gradually transition to adult stages through P17-P21 (Agbulut et al., 2003). Additionally, humans do not express a “true” type IIb myofiber, unlike mice (Smerdu et al., 1994). Therefore, the P17-21 mouse neuromuscular development resembles an early postnatal infant and the P44 mouse a sexually mature human adolescent. We have recently reviewed elsewhere (Wickramasekara and Stessman, 2019) the phenotypes of 34 unique individuals carrying putative disruptive (nonsense, frameshift, splice donor, or missense) variants in *KMT5B*. This included all publicly available individuals from the denovoDB database (Turner et al., 2017) (denovo-db, Seattle, WA), DECIPHER (Firth et al., 2009), and ClinVar (Landrum et al., 2018), as well as large-scale genetic screening publications (Yuen et al., 2000; De Rubeis et al., 2014; Iossifov et al., 2014; Homsy et al., 2015; Lelieveld et al., 2016; Turner et al., 2016; Jonsson et al., 2017; Yuen et al., 2017; Guo et al., 2018). There was not a highly penetrant undergrowth phenotype among *KMT5B* patients like has been observed in the *Kmt5b* mouse models (Schotta et al., 2008; Wickramasekara et al., 2021). Further, some have even suggested a *KMT5B*-associated overgrowth syndrome including tall stature and macrocephaly in these patients (Faundes et al., 2017; Wang et al., 2021). However, height data were not provided for the parents of *KMT5B* patients meaning that we cannot calculate the contribution of genetic background to this phenotype. Also, head circumference is often not normalized to height such that we cannot distinguish relative macrocephaly from an overgrowth syndrome in these patients. Indeed, we have identified increased relative brain weight in males in our mouse model (Supplementary Figure S4H). The resolution of these subtle developmental phenotypes will require a larger cohort of deeply-phenotyped *KMT5B* patients. Finally, we cannot rule out differences between mouse and man, both genetic and societal. Particularly in industrialized countries, failure to thrive in children is detected and treated early such that the natural progression of disease is never actualized. As we have reported previously, a conservative 38% of reported *KMT5B* patients had confirmed motor phenotypes (e.g., hypotonia) and/or global developmental delay, which often includes motor delays. For many individuals, data regarding motor development are never published. Indeed, motor deficits are thought to be vastly underreported among autists (Bhat, 2020). Given the rate of motor deficits and delays among the *KMT5B* patient population, the muscle phenotypes reported here, and work published by others (Neguembor et al., 2013; Boonsanay et al., 2016) we believe the mouse to be an important model for better understanding this patient population moving forward, including whether the same patients are affected by both motor and behavioral phenotypes.

In summary, this is the first study to evaluate the effects of *Kmt5b* loss on early muscle development within a genetic state and timeframe that are most relevant to humans carrying disruptive variation in *KMT5B*. We found that *Kmt5b* haploinsufficiency in mice results in significantly reduced skeletal muscle mass and neuromuscular strength underscored by increased NMJ fragmentation and an increased number of smaller type II myofibers. We conclude that KMT5B likely contributes to the process of muscle hypertrophy during early postnatal development, a mechanism that is perhaps independent from the adult quiescent muscle stem cell depletion phenotype associated with *Kmt5b* loss by others (Neguembor et al., 2013; Boonsanay et al., 2016). This is an exciting model as it suggests that some cases of neurodevelopmental motor delays may be the result of changes in skeletal muscle structure and maturation rather than the motor circuits in the brain.

## Data Availability

The original contributions presented in the study are included in the article/Supplementary Material, further inquiries can be directed to the corresponding author.

## Abbreviations

EDL, extensor digitorum longus; HET, heterozygous Kmt5b+/GT; NMJ, neuromuscular junction; SOL, soleus; TA, tibialis anterior; WT, wild-type Kmt5b+/+
