# Supplementary material for: KMT5B is required for early motor development
Source: Front Genet. 2022 Aug 12;13:901228. doi: 10.3389/fgene.2022.901228 (PMC9411648; doi:10.3389/fgene.2022.901228)
Supplement: Supplementary file 1 [file DataSheet1.PDF]

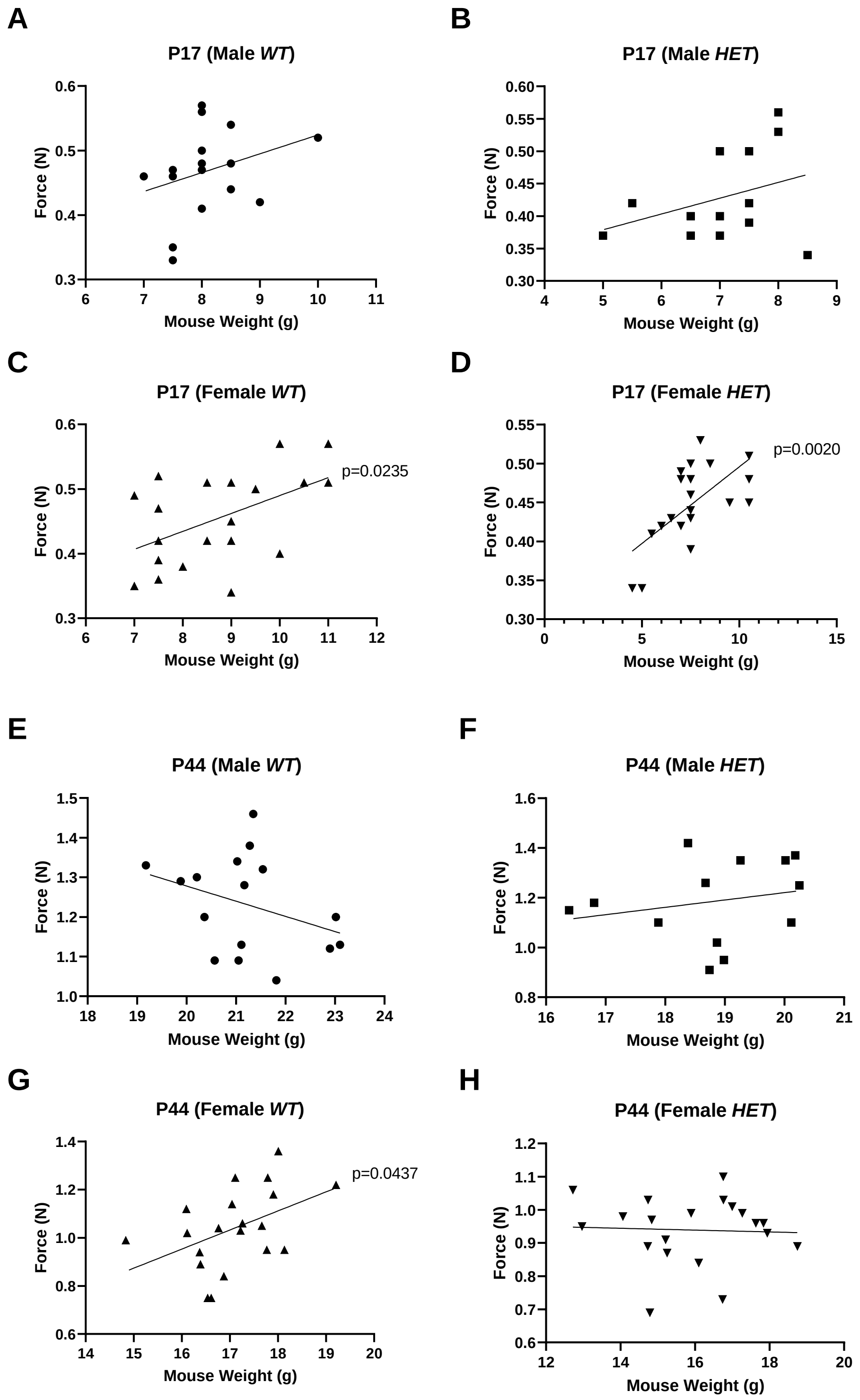

Figure S1. Neuromuscular strength and body weight are correlated in females but not males. Plots of mouse total body weight (g) and neuromuscular strength (force (N)) for each genotype and sex at P17 (**A-D**) and P44 (**E-H**). Pearson's correlation tests were performed for all sets of data presented; significant p-values are shown.

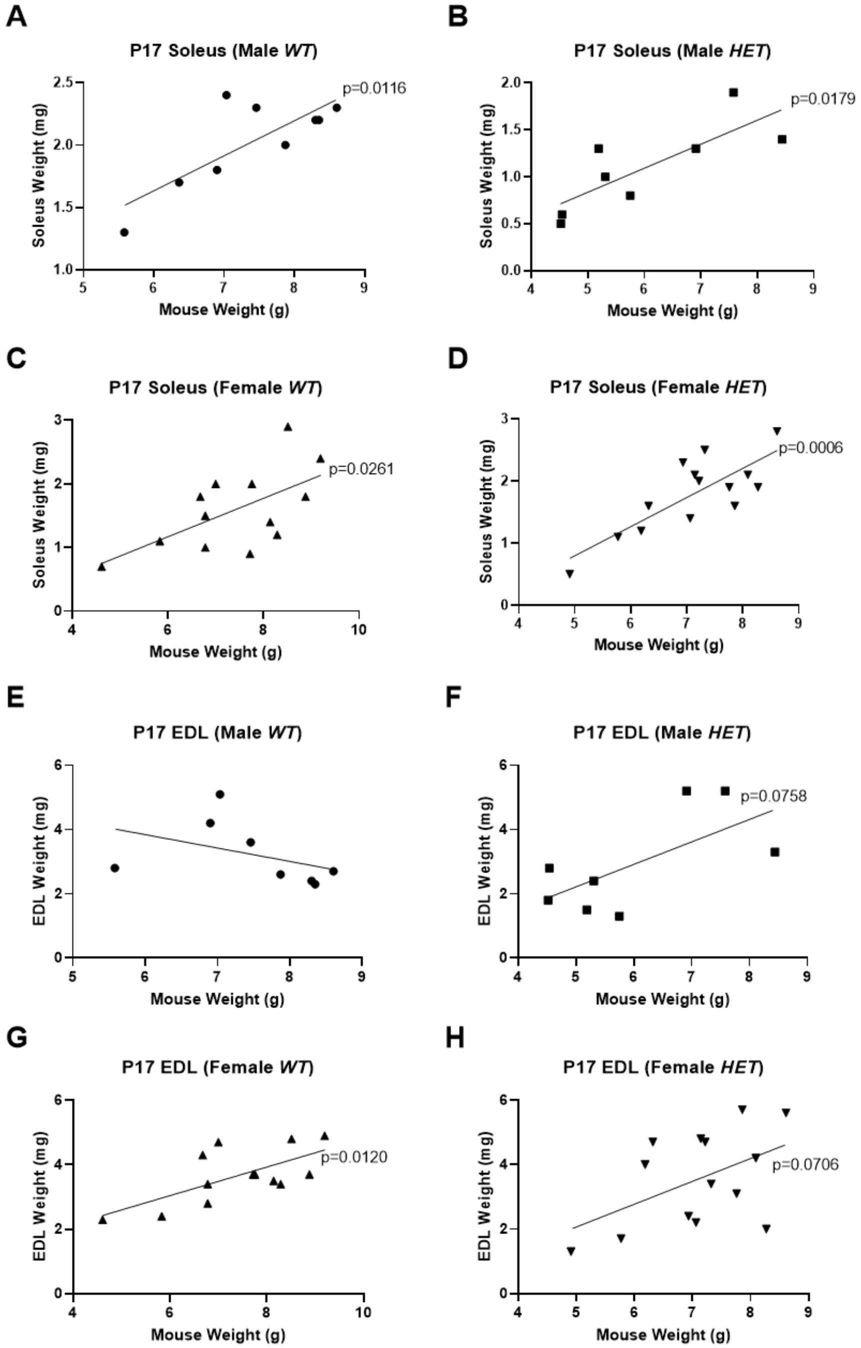

Figure S2. Skeletal muscle weight and body weight are largely positively correlated. Plots of mouse total body weight (g) and skeletal muscle weight (mg) for soleus and EDL for each genotype and sex. Significant positive correlation exists for (**A**) male *WT* soleus (p=0.0116; N=9), (**B**) male *HET* soleus (p=0.0179; N=8), (**C**) female *WT* soleus (p=0.0261; N=13), and (**G**) female *WT* EDL (p=0.0120; N=13). Correlations were not statistically significant for (**D**) female *HET* soleus (N=14), (**E**) male *WT* EDL (N=8), (**F**) male *HET* EDL (N=8), and (**H**) female *HET* EDL (N=14). Pearson's correlation tests were performed for all sets of data presented.

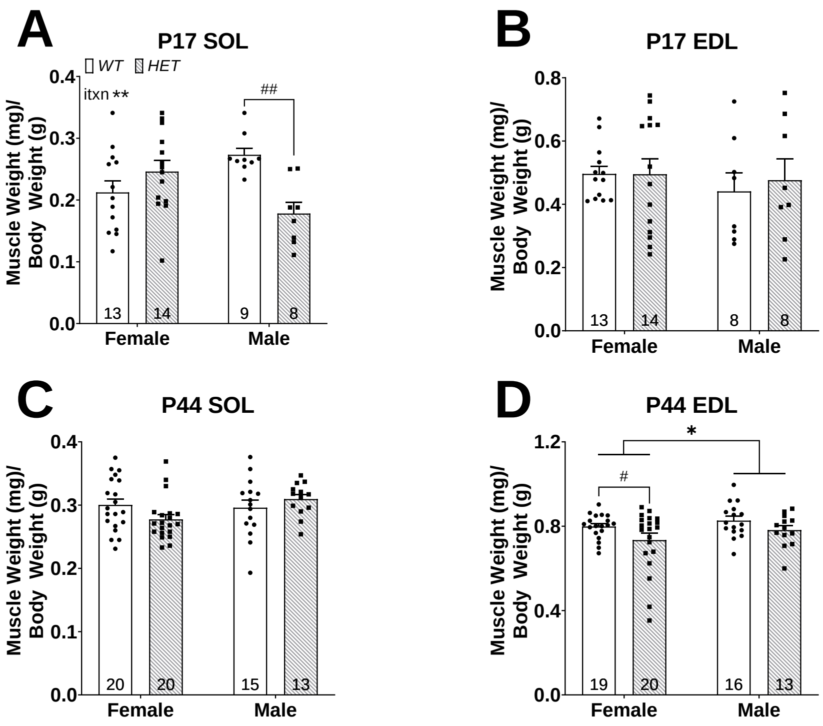

Figure S3. Decreased normalized muscle weights in *HET* mice. Graphs of soleus weight normalized to body weight (**A**,**C**), and EDL weight normalized to body weight (**B**,**D**) at P17 and P44 are shown. Normalized skeletal muscle weights were compared using 2-way ANOVA tests (independent variables: sex and genotype). *WT*: dots and open bars; *HET*: squares and grey hatched bars. *p < 0.05; **p < 0.01 (two-way ANOVA). #p < 0.05; ##p < 0.01 (post hoc test; effect of genotype only). Effects of sex or an interaction (itxn) between sex and genotype by 2-way ANOVA testing are shown as text in the upper left of the panel. Error bars show ± SEM; number of biological replicates are shown within each bar.

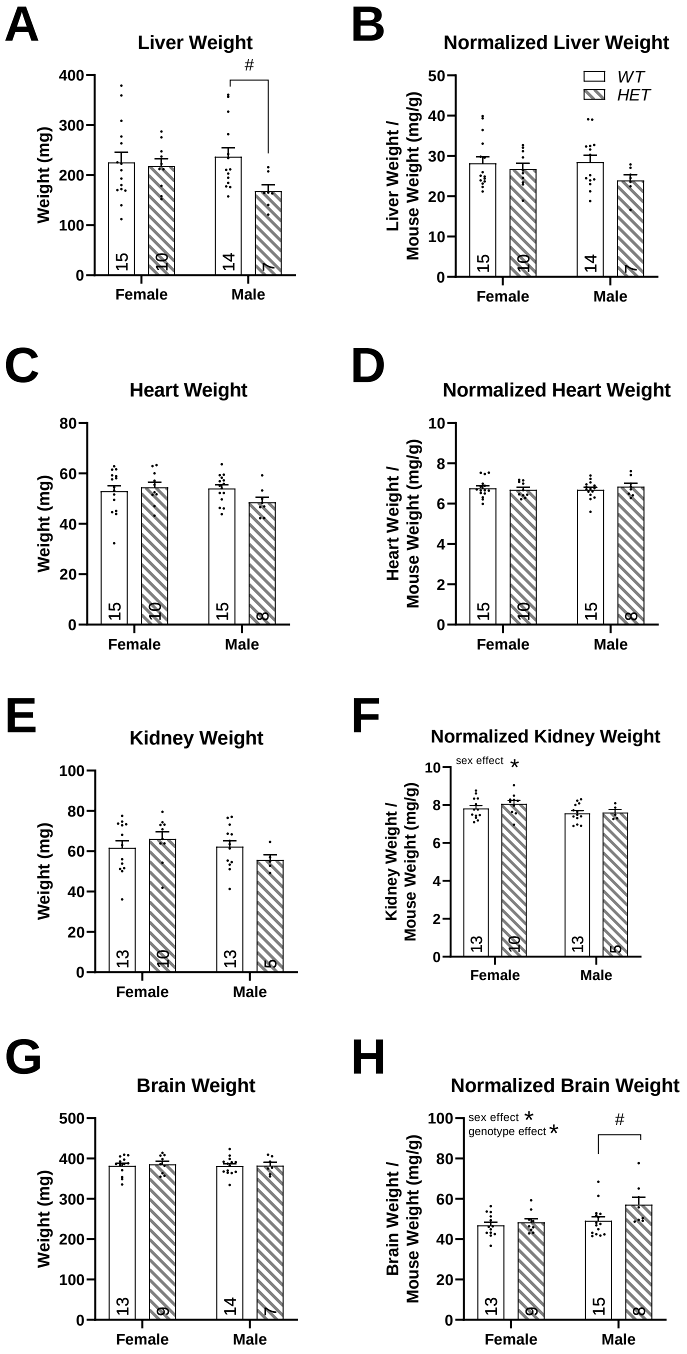

Figure S4. Organ weights in *HET* and *WT* mice. Graphs of organ weights shown as raw (**A, C, E, G**) and normalized to body weight (**B**, **D, F, H**) at P17. Data were compared using 2-way ANOVA tests (independent variables: sex and genotype). *WT*: dots and open bars; *HET*: squares and grey hatched bars. *p < 0.05 (two-way ANOVA). #p < 0.05 (post hoc test; effect of genotype only). Effects of sex or an interaction (itxn) between sex and genotype by 2-way ANOVA testing are shown as text in the upper left of the panel. Error bars show ± SEM; number of biological replicates are shown within each bar.

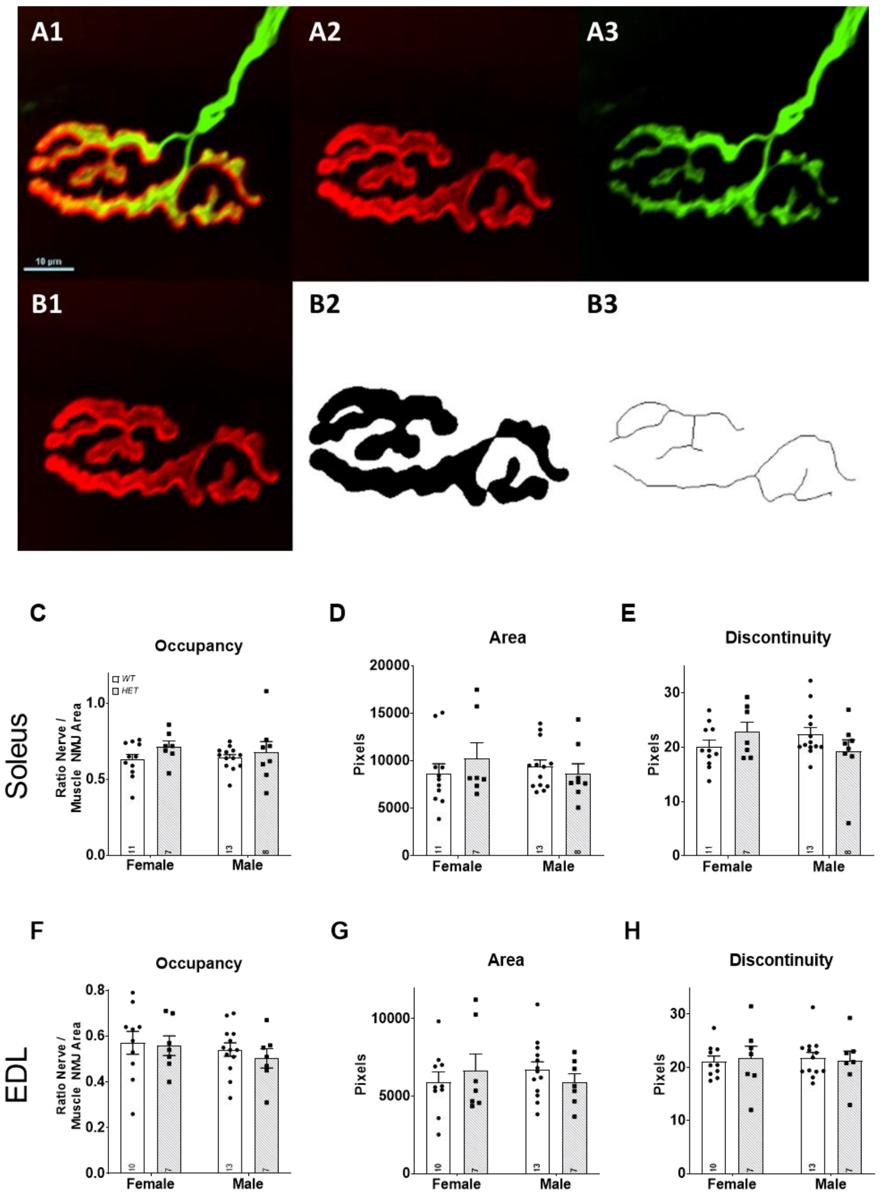

Figure S5. Expanded NMJ immunofluorescence results. Representative images of whole mount immunofluorescence staining for both nerve/vesicle and skeletal muscle portions of the NMJ (**A**) are displayed with combined image (**A1**), nicotinic receptors (skeletal muscle portion; **A2**), and synaptic vesicle and nerve filament (**A3**). Representative images of ImageJ processing results are show (**B**) with a single channel pre-processed image (**B1**), binary image for collecting area (**B2**), and skeletonized image for collecting connectivity parameters (**B3**). Imaging was achieved at 40X magnification. Results for neural filament and synaptic vesicle specific imaging are displayed on graphs (see Figure 2 for skeletal muscle portion results). Graphs show occupancy (**C**, **F**), total area (**D**, **G**), and calculated discontinuity (**E**, **H**) for soleus (**C**-**E**) and EDL (**F**-**H**) skeletal muscles. Y-axes of graphs are numbers of pixels. *WT*: dots and open bars; *HET*: squares and grey hatched bars. Statistical tests were 2-way ANOVA (independent variables genotype and sex). Error bars show ± SEM; number of biological replicates are shown within each bar.

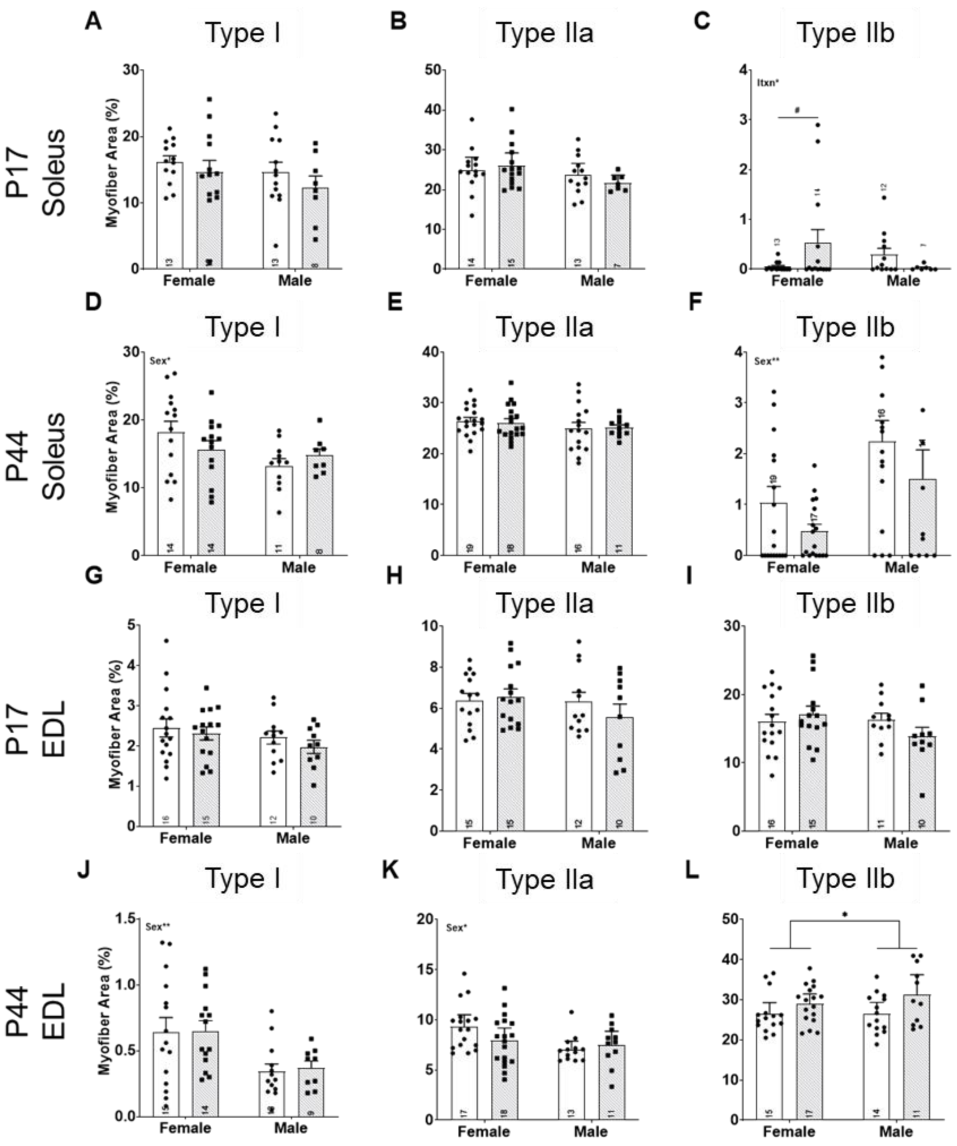

Figure S6. Myofiber area as a percentage of muscle cross sectional area. For myofiber types (IIb, IIa, and I), myofiber area was recorded using immunofluorescence specific to each myofiber type. Data were normalized to myofiber cross section areas (i.e., % of total area for each myofiber type). Graphs show results of each myofiber type in males and females from P17 soleus (**A**-**C**), P44 soleus (**D**-**F**), P17 EDL (**G**-**I**), and P44 EDL (**J**-**L**). *WT*: dots and open bars; *HET*: squares and grey hatched bars. *$p < 0.05$; **$p < 0.01$ (2-way ANOVA). Effects of sex or an interaction (itxn) between sex and genotype by 2-way ANOVA testing are shown as text in the upper left of the panel. Error bars show ± SEM; number of biological replicates are shown within each bar.

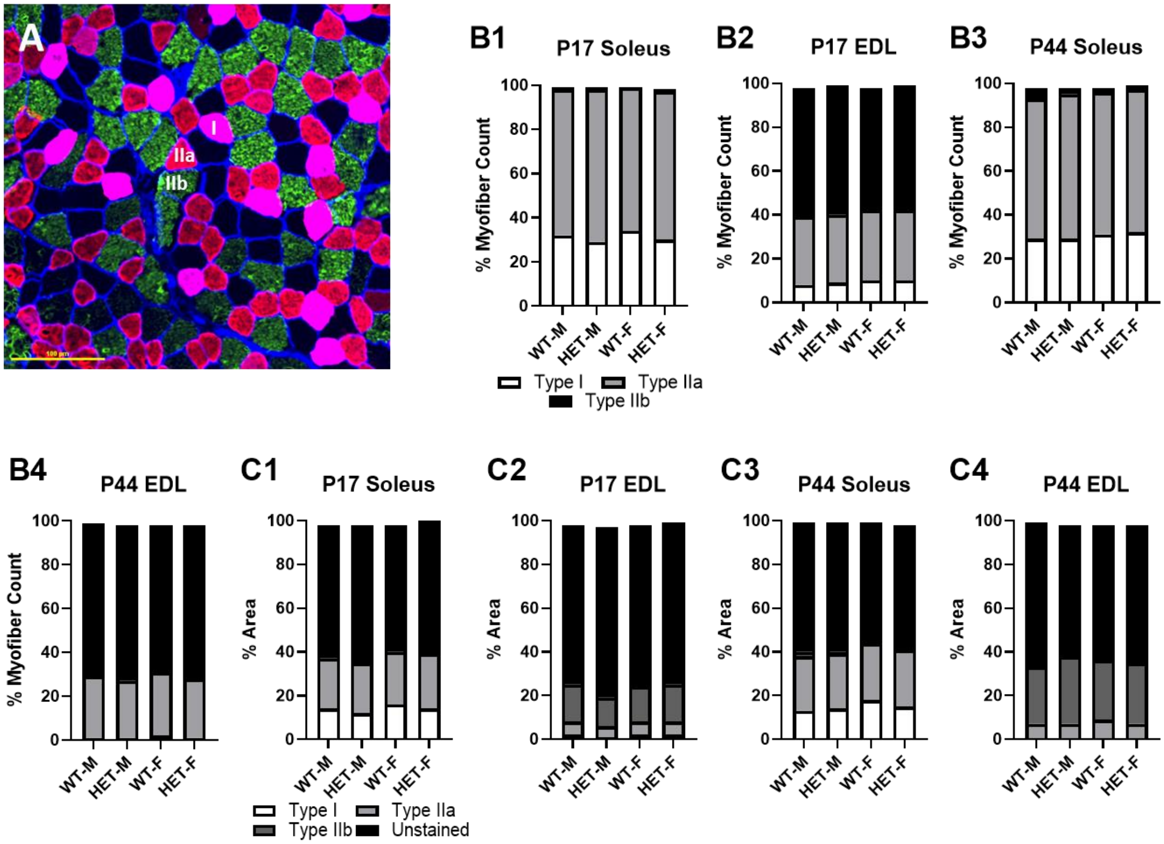

Figure S7. Relative ratios of each myofiber type by genotype and sex. Representative image of myosin heavy chain immunofluorescence (**A**) on a skeletal muscle section is shown. Imaging was achieved at 20X magnification. In image provided, type I appear pink, type IIa appear red, and type IIb appear green. Stacked graphical representation of data from Figure 3 and Figure S4. Myofiber count graphs (**B1**, **B2**, **B3**, **B4**) are normalized to the total number of myofibers counted. Percent muscle area graphs (**C1**, **C2**, **C3, C4**) are normalized to the muscle section cross sectional area. Chi-square tests showed no statistically significant differences between groups.

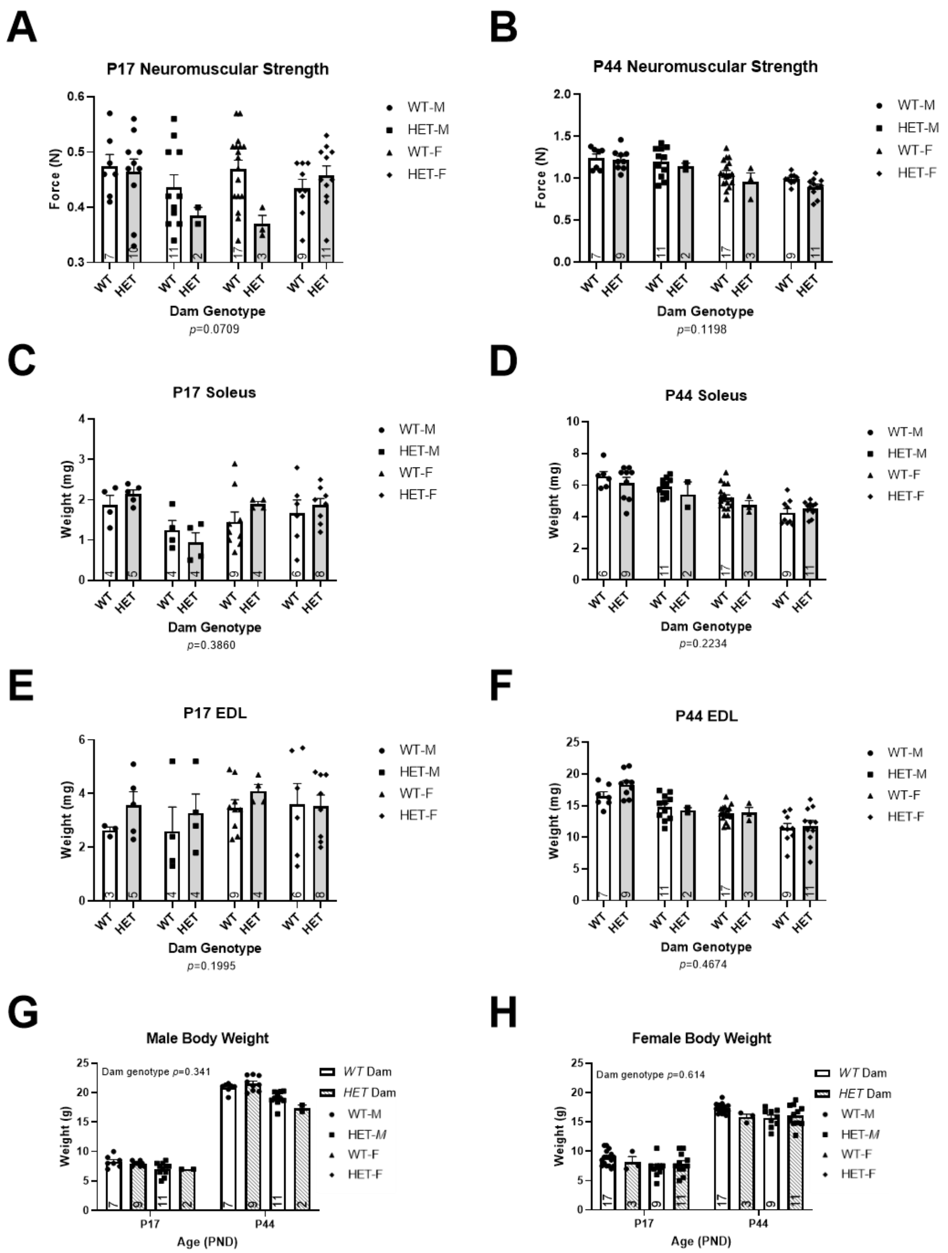

Figure S8. Genotype of the dam does not affect progeny performance. Differences in neuromuscular strength and skeletal muscle weights due to dam genotype were compared using 3-way ANOVAs (independent variables: mouse genotype, dam genotype, sex). Resulting graphs for (**A**) P17 neuromuscular strength, (**B**) P44 neuromuscular strength, (**C**) P17 soleus weight, (**D**) P44 soleus weight, (**E**) P17 EDL weight, and (**F**) P44 EDL weight show no difference between *WT* and *HET* dams. Differences in body weight due to dam genotype were compared using 3-way ANOVAs (independent variables mouse genotype, dam genotype, age) for each sex separately. Graphs depicting body weight (y-axis) and age (x-axis) show no difference between *WT* and *HET* dams in (**G**) male body weight, (**H**) female body weight. Error bars show ± SEM; number of biological replicates are shown within each bar.
